# The complete chloroplast genome of *Dicliptera tinctoria* (Nees) Kostel. and comparative analysis of chloroplast genomes in Acanthaceae

**DOI:** 10.1590/1678-4685-GMB-2023-0297

**Published:** 2024-06-14

**Authors:** Thi Thanh Nga Le, Minh Thiet Vu, Hoang Dang Khoa Do

**Affiliations:** 1 Nguyen Tat Thanh University, NTT Hi-Tech Institute, Ho Chi Minh City, Vietnam

**Keywords:** Acanthaceae, genomic variation, molecular markers, nucleotide diversity, simple sequence repeats

## Abstract

*Dicliptera tinctoria* is a member of Acanthaceae, which has a wide distribution and contains potentially medicinal species, and exhibited pharmaceutical potentials. This study sequenced and characterized the complete chloroplast genome of *Dicliptera tinctoria*. The newly sequenced cpDNA of *D. tinctoria* was 150,733 bp in length and had a typical quadripartite structure consisting of a large single copy (LSC, 82,895 bp), a small single copy (SSC, 17,249 bp), and two inverted repeat (IRs, 25,295 bp each) regions. This genome also contained 80 protein-coding genes, 30 transfer RNAs, and four ribosomal RNAs, which is identical to other chloroplast genomes in Acanthaceae family. Nucleotides diversity analysis among chloroplast genomes of Acanthaceae species revealed eight hypervariable regions, including *trnK_UUU-matK*, *trnC_GCA-petN*, *accD*, *rps12-clpP*, *rps3-rps19*, *ycf1-ndhF*, *ccsA-ndhD*, and *ycf1.* Phylogenetic analysis revealed the paraphyly of *Dicliptera* species and monophyly in four Acanthaceae subfamilies. These results provide an overview of genomic variations in Acanthaceae chloroplast genome, which is helpful for further genomic studies.

*Dicliptera tinctoria* (Nees) Kostel. is a member of Acanthaceae, a flowering plant family of more than 3,500 species divided into 228 genera with diverse morphology, characteristics, and geographical distribution (POWO, 2023). Acanthaceae family formally comprised of four subfamilies, including Acanthoideae (217 genera, 3220 species), Avicennioideae (one genus, eight species), Nelsonioideae (five genera, 180 species), and Thunbergioideae (five genera, 190 species) (POWO, 2023). Previous studies revealed phytochemical and pharmacological aspects of the Acanthaceae species such as antioxidant, antibacterial, antifungal, and anti-inflammatory (Gangaram et al., 2021). The extract of *D. tinctoria* exhibited potential features for anti-snake venoms, natural dyeing products, and antibacterial agents (Adrianta, 2021).

The chloroplast genome (cpDNA) is an essential component of chloroplast in land plants and contains photosynthesis-related genes (Daniell et al., 2016). The chloroplast genome data provide useful information for conducting phylogeny, molecular markers, and population genetics (Daniell *et al*., 2016). In Acanthaceae, chloroplast genomes of some species, such as *Avicennia marina*, *Aphelandra knappiae*, *Peristrophe japonica*, *Barleria prionitis*, *Strobilanthes biocullata* and *Justicia* species, were sequenced and described (Chen et al., 2021; Niu et al., 2023). Previously, complete cpDNAs of *Dicliptera* species, including *D. montana*, *D. acuminata*, *D. peruviana*, *D. ruiziana*, and *D. mucronata* were reported, but that of *D. tinctoria* has not yet been characterized (Huang et al., 2020).

In this study, the complete *Dicliptera tinctoria* chloroplast genome was sequenced and characterized. Additionally, the cpDNAs of Acanthaceae species were collected and used for comparative analysis, revealing different hypervariable regions, repeat contents, and boundaries between four regions of chloroplast genomes. Additionally, analysis of the phylogenetic relationship of 49 species of Acanthaceae was also conducted. The results of the current study provide useful data for further studies on genomic evolution and population genetics of Acanthaceae members.

Fresh leaves of *Dicliptera tinctoria* were collected at Saigon Hi-tech Park, Ho Chi Minh City, Vietnam (10°50’20.2”N, 106°49’04.9”E) and dried using silica gel beads. The specimen of *D. tinctoria* was kept at NTT Hi-Tech Institute of Nguyen Tat Thanh University, Vietnam, under voucher number NNTU-20221023-P012. Total genomic DNA was extracted from dried leaves using the cetyltrimethylammonium bromide (CTAB) method (Doyle and Doyle, 1987). The extracted DNA samples were qualified using gel electrophoresis and NanoDrop One spectrophotometer (Thermo Fisher Scientific, USA). For next-generation sequencing, a high-quality DNA sample (showing a clear band on agarose gel, concentration above 100 ng/ul, and having ratios of A260/280 and A260/230 ranging from 1.8 to 2.0 and 2.0 to 2.2, respectively) was used. The sequencing library was prepared with a TruSeq Nano DNA Sample Preparation Kit (Illumina, USA) before being sequenced on the Illumina MiSeq platform which generated paired-end reads of 151 bp. The raw data (3,317,424 reads) were qualified and filtered using FastQC v0.12.1 and Trimmomatic v0.32 program to remove low-quality reads (Q score < 20), adapters, N-containing reads, and short reads (< 100 bp) (Andrews, 2010; Bolger et al., 2014). The qualification results showed that all reads had good quality (Q score ˃ 20, no reads containing N and adapters, and no short reads). The cpDNA of *D. tinctoria* was assembled using NOVOPlasty v4.3.3 with the complete chloroplast genome of *Dicliptera montana* (Accession number MK833946) as the seed and reference sequence (Dierckxsens et al., 2016). There were 722,236 out of 3,317,424 reads that assembled the complete chloroplast genome of *D. tinctoria*. The gene content of *D. tinctoria* chloroplast genome was annotated using Geseq programs with default setting (Tillich et al., 2017). The complete chloroplast genome of *D. tinctoria* (average coverage depth = 756x) was deposited to GenBank (https://www.ncbi.nlm.nih.gov/) under the accession number OR063946. The map of cpDNA of *D. tinctoria* was depicted using the OGDRAW program (Greiner et al., 2019).

A total of 37 complete chloroplast genomes were retrieved from GenBank database (https://www.ncbi.nlm.nih.gov/) and used for further studies (Table S1). The boundaries between LSC, SSC, and IR regions were identified using IRscope (Amiryousefi et al., 2018). To determine nucleotide diversity across 37 species of Acanthaceae, DnaSP 6 was used to calculate the pi values with the parameters of sliding window at 2000 and step size at 100 (Rozas et al., 2017). 

The REPuter program was used to find tandem repeats with a minimum length of 20 bp and to identify the type of repeats including reverse, forward, complement, and palindromic repeats (Kurtz, 2001). The Phobos program (embedded in Genenious Prime) was used to identify simple single repeats (SSRs) by indicating mono-, di-, tri-, tetra, penta-, hexanucleotides with repeat threshold settings of 10, 5, 4, 3, 3, and 3, respectively (Mayer, 2006). 

The complete chloroplast genomes of 49 Acanthaceae (Table S1) and two outgroups of *Sesamum indicum* (GenBank accession number NC_016422) and *Torenia violacea* (GenBank accession number NC_072147) were aligned using MUSCLE embedded in Geneious Prime 2022.2 (Edgar, 2004). The aligned sequences were used to reconstruct the phylogenetic tree using IQ-TREE web server with auto-detection of substitution model (identified as TVM+F+R5) and 1000 bootstrap replicates (Trifinopoulos et al., 2016). For Bayesian inference analysis, Mrbayes v3.2.7a was used with TVM+I+G (Akaike information criterion) model resulted from jModeltest 2 (Ronquist et al., 2012; Darriba et al., 2012). A total of 1,000,000 generations were run that show a split frequency lower than 0.01. Additionally, 25% of the sampled tree was discarded. The phylogenetic tree was illustrated using Figtree v1.4.4 (http://tree.bio.ed.ac.uk/software/figtree/).

The cpDNA of *D. tinctoria* was 150,733 bp long and had a typical quadripartite structure containing a large single copy (LSC, 82,895 bp) and a small single copy (SSC, 17,249 bp), separated by two inverted repeat (IRs, 25,295 bp each) regions (Figure 1). All 37 surveyed cpDNAs of Acanthaceae also possessed the tetrad structure with a total length ranging from 143,016 bp (*Ruellia brittoniana*) to 153,783 bp (*Staurogyne concinnula)* (Table S2). The GC contents of the examined cpDNA sequences varied from 38.0% to 38.7%. Among the three regions, the two IR regions took the most prominent part, with CG content ranging from 43.1% to 46%, while LSC and SSC regions accounted for smaller amount ranging from 35.9% to 37% and from 31.8% to 33%, respectively. Despite the different sizes of the LSC, SSC, and IR regions, most cpDNA possessed 80 protein-coding genes, 30 tRNAs, and four rRNAs (Table 1). However,*Avicennia marina*(NC_047414) had 79 protein-coding genes due to the lack of the*psaI*gene. 

**Figure 1 -:**
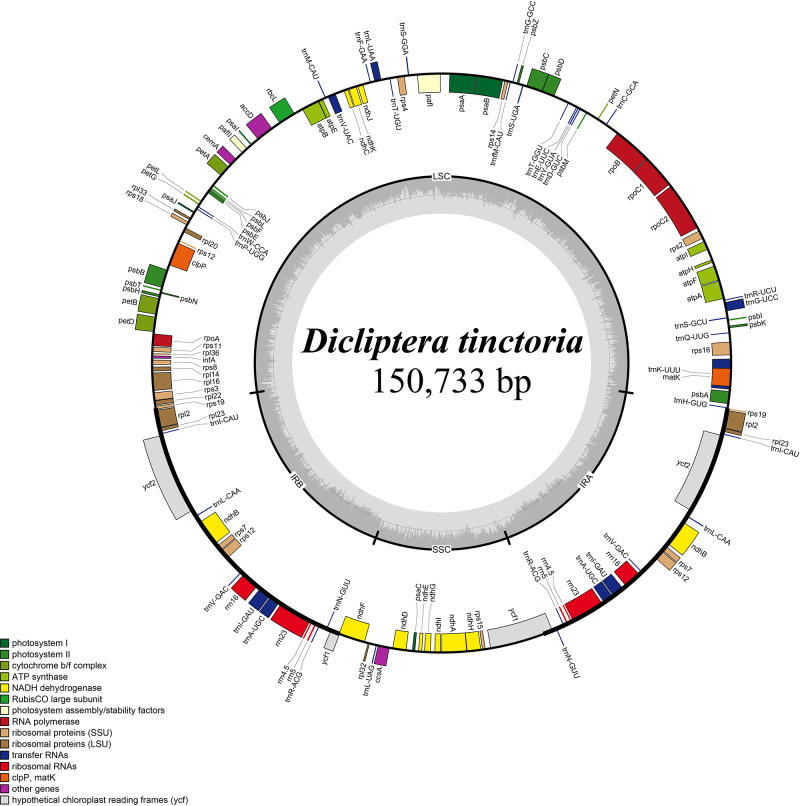
Map of the *D. tinctoria* chloroplast genome. Genes shown inside the circle are transcribed clockwise, and those outside the circle are counterclockwise transcribed. The light grey and the darker grey in the inner circle correspond to AT and GC content, respectively. Colors indicate different functional groups. LSC: large single copy; SSC: small single copy; IRA/IRB: Inverted repeat regions.

**Table 1 -:** List of genes in the plastome of *D. tinctoria*.

Groups of genes	Name of genes
Ribosomal RNAs	*rrn4.5(2x), rrn5(2x), rrn16(2x), rrn23(2x)*
Transfer RNAs	trnA_UGC*(2x), trnC_GCA, trnD_GUC, trnE_UUC, trnF_GAA, trnG_UCC*, trnG_GCC, trnH_GUG(2x), trnI_CAU(2x),trnI_GAU*(2x), trnK_UUU*, trnL_UAA*, trnL_UAG, trnL_CAA(2x), trnfM_CAU, trnM_CAU, trnN_GUU(2x), trnP_UGG, trnQ_UUG, trnR_UCU, trnR_ACG(2x), trnS_GCU, trnS_UGA, trnS_GGA, trnT_GGU, trnT_UGU, trnV_UAC*, trnV_GAC, trnW_CCA, trnY_GUA
Photosystem I	*psaA, psaB, psaC, psaI*§, psaJ, pafI*,pafII
Photosystem II	*psbA, psbB, psbC, psbD, psbE, psbF, psbH, psbI, psbJ, psbK, psbL, psbM, psbN, psbT, psbZ*
Cytochrome	petA, petB*, petD*, petG, petL, petN
ATP synthases	atpA, atpB, atpE, atpF*, atpH, atpI
Large unit of Rubisco	*rbcL*
NADH dehydrogenase	ndhA*, ndhB*(2x), ndhC, ndhD, ndhE, ndhF, ndhG, ndhH, ndhI, ndhJ, ndhK
ATP_dependent protease subunit P	clpP*
Envelop membrane protein	*cemA*
Large units of ribosome	rpl2*(2x), rpl14, rpl16*, rpl20, rpl22(2x), rpl23(2x), rpl32, rpl33, rpl36
Small units of ribosome	rps2, rps3(2x), rps4, rps7(2x), rps8, rps11, rps12*(2x), rps14, rps15, rps16*, rps18, rps19(2x)
RNA polymerase	rpoA, rpoB, rpoC1*, rpoC2
Initiation factor	*infA*
Miscellaneous protein	*accD, ccsA, matK*
Hypothetical proteins and conserved reading frames	*ycf1, ycf2(2x)*

Gene* = gene with introns; Gene(2x) = duplicated gene in IR region. Gene§ = gene was deleted in *Avicennia marina*.

The junctions among LSC, SSC, and IR regions in 37 chloroplast genomes of Acanthaceae species were primarily located in the intergenic region (IGS). Specifically, the LSC-IRa boundary was mostly identified in the IGS before *trnH_GUG*(Figure S1, Table S2). The LSC-IRb junction was located between*ycf2*and*psbA*, caused by the rearrangement of *psbA* from LSC to IR regions. However, the LSC-IRb junction of *R. brittoniana*,*Strobilanthes*, and*Avicenia*taxa was located within*ycf2*. In Acanthaceae chloroplast genomes, the boundaries between SSC and IR regions were found within *ycf1*and*ndhF*genes, which commonly overlapped at the junction, except in*Clinacanthus nutans* (Figure S1, Table S2).

The nucleotide divergence analysis showed that pi values ranged from 0.01693 to 0.10356 in LSC and from 0.04789 to 0.14377 in SSC. While in IR regions, pi values varied between 0.00323 and 0.03942 (Figure S2). The most nucleotide variable regions were *trnK*_UUU - *matK* (0.10356), *trnC*_GCA - *petN* (0.9164), *accD* (0.8453), *rps12* - *clpP* (0.7454), *rps3* - *rps19* (0.07762),*ndhF* (0.11004), *ccsA* - *ndhF* (0.10225), and *ycf1* (0.14377) (Figure S2).

The SSR analysis of 37 Acanthaceae cpDNAs revealed that mononucleotides were dominant (accounting for 55.6%), followed by tetranucleotides at 17.9%, while dinucleotides and tronucleotides accounted for 11.7% and 11.6%, respectively (Figure S3). Pentanucleotides and hexanucleotides were rarely found, scoring at 1.6% and 1.1%, respectively. The mononucleotide repeat, which ranged from 79 in *A. officinalis* to nine in *B. cilaris*, was the only type present in all species. Notably,*A. officinalis*had the highest num ber of repeats (106 repeats), while *Blepharis ciliaris* had the lowest number of SSRs (24 repeats) (Figure S4). There were 547 long repeats consisting of forward and palindromic types among cpDNAs of Acanthaceae (Figure S5). Reverse and complement repeats were not found in surveyed species of Acanthacae. *C. nutan* and *P. haikangenes* had the largest number of repeats at 54 and 51, respectively. In addition, *Dicliptera* species had fewer repeats, ranging from 8 to 11. Most repeats were 20-29 bp in length (Figure S5).

The phylogenetic analysis revealed the monophyly of Acanthaceae with high support values (bootstrap value = 100 and posterior probability = 1) (Figure 2). Furthermore, the Nelsonioideae subfamily is a basal clade of Acanthaceae. Meanwhile, Thunbergioideae and Avicennioideae formed a clade, which is a sister to Acanthoideae. Among surveyed species, *Echinacanthus* and *Justicia* taxa exhibit a paraphyletic group in Acanthaceae. Similarly*, Dicliptera* species formed a polyphyletic group that included *Peristrophe flava* and *Justicia* species (Figure 2).

**Figure 2 -:**
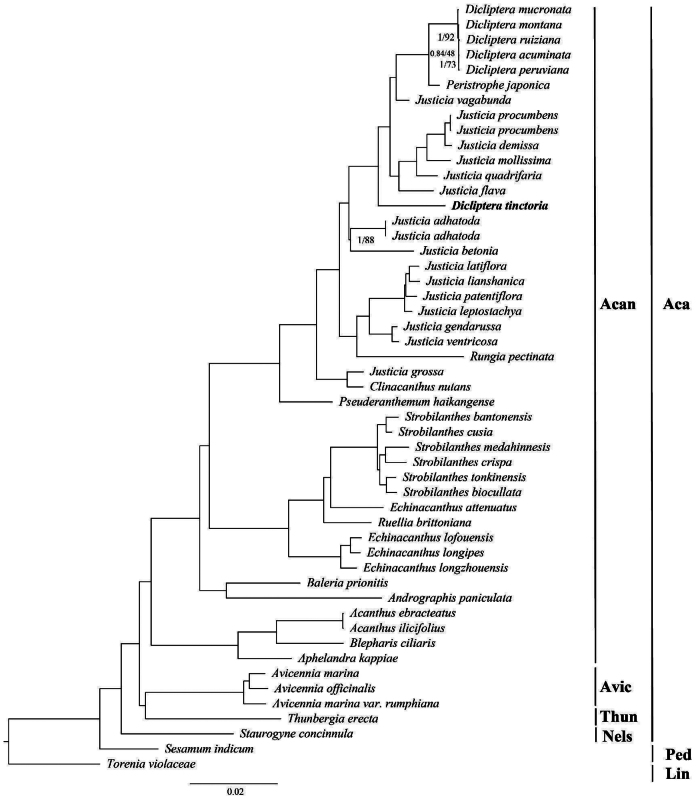
Phylogenetic tree of Acanthaceae inferred from complete chloroplast genomes using Maximum likelihood and Bayesian Inference methods. The bold name indicates the newly sequenced chloroplast genome in Acanthaceae. The Posterior probability < 1 and bootstrap values < 100 are shown at each node. Acan: Acanthoideae; Thun: Thunbergioideae; Avic: Avicenioideae; Nels: Nelsonioideae; Aca: Acanthaceae; Ped: Pedaliaceae; Lin: Linderniaceae.

In this study, the cpDNA sequences of *D. tinctoria* was sequenced and characterized. Comparative analyses revealed different hypervariable regions, repeat content, and dynamic boundaries among LSC, SSC, and IR regions. The genomic information provided essential data for further studies on genetic population, molecular markers, and evolutionary history of *Dicliptera* genus and related taxa. Phylogenetic analysis indicated the paraphyly of *Dicliptera* species, suggesting new circumscription of *Dicliptera* within Acanthoideae. Therefore, further studies that cover more members of *Dicliptera* should be conducted to explore deeper phylogenetic relationships within the Acanthaceae genera.

## Supplementary material

The following online material is available for this article:

Table S1 - List of complete chloroplast genomes in Acanthaceae used in this study.

Table S2 - Features of surveyed chloroplast genomes in Acanthaceae.

Figure S1 - The junctions among LSC, SSC, and IR regions in thirty-seven chloroplast genome sequences of Acanthaceae.

Figure S2 - Nucleotide diversity of thirty-seven cpDNA sequences of Acanthaceae.

Figure S3 - The proportion of different types of simple sequence repeats in Acanthaceae.

Figure S4 - The number of SSR found in chloroplast genomes of Acanthaceae species. The blue line revealed the total number of each species; the type of SSRs was represented by the color column on the right.

Figure S5 - The number of two types of long repeats among cpDNA of Acanthaceae. P stands for palindromic; F stands for forward; the green line represents the total number of both forward and palindromic repeats of Acanthaceae.
