# Supplementary material for: The complete chloroplast genome of Dicliptera tinctoria (Nees) Kostel. and comparative analysis of chloroplast genomes in Acanthaceae
Source: Genet Mol Biol. 2024 Jun 14;47(2):e20230297. doi: 10.1590/1678-4685-GMB-2023-0297 (PMC11182309; doi:10.1590/1678-4685-GMB-2023-0297)
Supplement: Table S1 - [file 1415-4757-GMB-47-2-e20230297-s1.pdf]

Genetics and Molecular Biology
DOI: https://doi.org/10.1590/1678-4685-GMB-2023-0297

Supplementary Material to "The complete chloroplast genome of Dicliptera tinctoria (Nees) Kostel. and comparative analysis of chloroplast genomes in Acanthaceae"

Table S1 - List of complete chloroplast genomes in Acanthaceae used in this study.

No.	Species names	Accession number	No.	Species names	Accession number
1	*Acanthus ebracteatus*	MT240944	20	*Echinacanthus longzhouensis*	NC_039678
2	*Acanthus ilicifolius*	NC_054308	21	*Justicia adhatoda*	NC_047476
3	*Andrographis paniculata*	NC_022451	22	*Justicia flava*	NC_044862
4	*Aphelandra knappiae*	NC_041424	23	*Justicia leptostachya*	NC_044668
5	*Avicennia marina*	NC_047414	24	*Justicia procumbens*	NC_062458
6	*Avicennia marina* var. *rumphiana*	NC_061403	25	*Justicia ventricosa*	NC_059953
7	*Avicennia officinalis*	NC_063736	26	*Peristrophe japonica*	NC_056198
8	*Barleria prionitis*	NC_048478	27	*Pseuderanthemum haikangense*	NC_057986
9	*Blepharis ciliaris*	NC_046601	28	*Ruellia brittoniana*	MW697905
10	*Clinacanthus nutans*	NC_042162	29	*Rungia pectinata*	MK946456
11	*Dicliptera acuminata*	MK830556	30	*Staurogyne concinnula*	NC_064996
12	*Dicliptera montana*	MK833946	31	*Strobilanthes bantonensis*	MT576695
13	*Dicliptera mucronata*	MK848596	32	*Strobilanthes biocullata*	NC_060347
14	*Dicliptera peruviana*	MK833945	33	*Strobilanthes crispa*	NC_060385
15	*Dicliptera ruiziana*	MK833947	34	*Strobilanthes cusia*	NC_037485
16	*Dicliptera tinctoria*	OR063946	35	*Strobilanthes medahinnensis*	NC_058776
17	*Echinacanthus attenuatus*	NC_039762	36	*Strobilanthes tonkinensis*	NC_059902
18	*Echinacanthus lofouensis*	NC_035876	37	*Thunbergia erecta*	MZ555773
19	*Echinacanthus longipes*	NC_039761	**38**	***Justicia quadrifaria***	**MN848243**
**39**	***Justicia betonia***	**MN848244**	**40**	***Justicia procimbens***	**MN848245**
**41**	***Justicia latiflora***	**MN848246**	**42**	***Justicia mollissima***	**MN848247**
**43**	***Justicia patentiflora***	**MN848248**	**44**	***Justicia adhatoda***	**MN848249**
**45**	***Justicia vagabunda***	**MN848250**	**46**	***Justicia grossa***	**MN848251**
**47**	***Justicia gendarussa***	**MN848252**	**48**	***Justicia demissa***	**MN885664**
**49**	***Justicia lianshanica***	**MN885665**			

The bold format indicates samples for phylogenetic analysis.
