# Supplementary material for: The complete chloroplast genome of Dicliptera tinctoria (Nees) Kostel. and comparative analysis of chloroplast genomes in Acanthaceae
Source: Genet Mol Biol. 2024 Jun 14;47(2):e20230297. doi: 10.1590/1678-4685-GMB-2023-0297 (PMC11182309; doi:10.1590/1678-4685-GMB-2023-0297)
Supplement: Table S2 - [file 1415-4757-GMB-47-2-e20230297-s2.pdf]

Genetics and Molecular Biology
DOI: https://doi.org/10.1590/1678-4685-GMB-2023-0297

Supplementary Material to "The complete chloroplast genome of Dicliptera tinctoria (Nees) Kostel. and comparative analysis of chloroplast genomes in Acanthaceae"

Table S2 - Features of surveyed chloroplast genomes in Acanthaceae.

Species	Length (bp)	LSC length (bp)	SSC length (bp)	IR length (bp)	Gene content (Protein /tRNA/ rRNA)	CG content	CG content (LSC)	CG content (SSC)	CG content (IR)	Junction	
										**LSC/IR**	**SSC/IR**
Acanthus ebracteatus	150,823	82,028	17,191	25,302	80/30/4	38.4	36.4	32.3	43.7	IGS (*rps19/rpl2*)	*Overlap 31 bp;* *ycf1* (817 bp)
Acanthus ilicifolius	150,758	82,963	17,191	25,302	80/30/4	38.4	36.4	32.3	43.7	IGS (*rps19/rpl2*)	*Overlap 31 bp;* *ycf1* (817 bp)
Andrographis paniculata	150,249	82,459	17,110	25,340	80/30/4	38.3	36.4	31.8	43.5	IGS (*rps19/rpl2*)	Overlap 40 bp; *ycf1* (983 bp)
Aphelandra knappiae	152,457	83,861	17,888	25,354	80/30/4	38.5	36.5	32.7	43.7	IGS (*rps9/rpl2*)	Overlap 41 bp; *ycf1* (768 bp)
Avicennia marina	147,909	88,331	17,766	20,906	79/30/4	38.5	35.9	32.9	44.3	*ycf2* (2283 bp)	Ovelap 8 bp; *ycf1* (761 bp)
Avicennia marina var. rumphiana	148,264	87,995	17,949	21,160	80/30/4	38.4	36.8	32.7	44.3	*ycf2* (1997 bp)	Overlap 7 bp; *ycf1* (757 bp)
Avicennia officinalis	148,209	87,965	17,878	21,183	80/30/4	38.4	36.8	32.8	44.3	*ycf2* (2014 bp)	Overlap 15 bp; *ycf1* (765 bp)
Barleria prionitis	152,217	83,772	17,803	25,321	80/30/4	38.3	36.4	32.6	43.5	IGS (*rps19/rpl2*)	Overlap 36 bp; *ycf1* (763 bp)
Blepharis ciliaris	149,717	82,057	16,998	25,331	80/30/4	38.5	36.6	32.5	43.7	IGS (*rps19/rpl2*)	Overlap 58 bp; *ycf1* (805 bp)
Clinacanthus nutans	151,669	83,502	17,303	25,432	80/30/4	38.4	36.5	32.4	43.5	IGS *(rps19/rpl2)*	Overlap 0 bp; *ycf1* (849 bp)
Dicliptera acuminata	150,738	82,844	17,092	25,401	80/30/4	38	36	31.9	43.3	*rps19* (102 bp)	Overlap 117 bp; *ycf1* (812 bp)
Dicliptera montana	150,689	82,796	17,091	25,401	80/30/4	38	36	31.9	43.3	*rps19* (102 bp)	Overlap 117 bp; *ycf1* (812 bp)
Dicliptera mucronata	150,720	82,834	17,084	25,401	80/30/4	38	36	31.9	43.3	*rps19* (102 bp)	Overlap 117 bp; *ycf1* (812 bp)
Dicliptera peruviana	150,811	82,919	17,090	25,401	80/30/4	38	36	31.9	43.3	*rps19* (102 bp)	Overlap 117 bp; *ycf1* (812 bp)

**Species**	**Length (bp)**	**LSC length (bp)**	**SSC length (bp)**	**IR length (bp)**	**Gene content (Protein /tRNA/ rRNA)**	**CG content**	**CG content (LSC)**	**CG content (SSC)**	**CG content (IR)**	**Junction**	
										**LSC/IR**	**SSC/IR**
Dicliptera ruiziana	150,750	82,843	17,091	25,408	80/30/4	38	36	31.9	43.3	*rps19* (102 bp)	Overlap 117 bp; *ycf1* (812 bp)
Dicliptera tinctoria	150,733	82,895	17,249	25,295	80/30/4	38.2	36.3	32.1	43.4	*rps19* (102 bp)	Overlap 75 bp; *ycf1* (802 bp)
Echinacanthus attenuatus	152,672	83,609	17,739	25,662	80/30/4	38.3	36.3	32.5	43.4	IGS (*rpl22/rps19*)	Overlap 55 bp; *ycf1* (797 bp)
Echinacanthus lofouensis	151,333	82,560	17,397	25,688	80/30/4	38.7	37	33	43.5	IGS (*rpl22/rps19*)	Overlap 69 bp; *ycf1* (797 bp)
Echinacanthus longipes	152,644	83,833	17,429	25,691	80/30/4	38.6	36.8	33	43.5	IGS (*rpl22/rps19*)	Overlap 73 bp; *ycf1* (800 bp)
Echinacanthus longzhouensis	152,385	83,946	17,571	25,434	80/30/4	38.6	36.8	32.8	43.6	*rps19* (97 bp)	Overlap 70 bp; *ycf1* (797 bp)
Justicia adhatoda	149,479	82,576	17,009	24,947	80/30/4	38.3	36.4	32.4	43.4	*rps19* (104 bp)	Overlap 129 bp; *ycf1* (821 bp)
Justicia flava	150,888	82,995	16,918	25,488	80/30/4	38.2	36.3	32.2	43.4	*rps19* (102 bp)	Overlap 117 bp; *ycf1* (812 bp)
Justicia leptostachya	149,227	82,114	16,977	25,068	80/30/4	38.2	36.2	32.4	43.4	*rps19* (85 bp)	Overlap 122 bp; *ycf1* (817 bp)
Justicia procumbens	150,454	82,404	16,952	25,549	80/30/4	38.3	36.4	32.7	43.3	*rps19* (102 bp)	Overlap 117 bp; ycf*1* (812 bp)
Justicia ventricosa	149,700	82,324	17,260	25,058	80/30/4	38.4	36.5	32.7	43.4	*rps19* (76 bp)	*Overlap* 121 bp*;* *ycf1* (819 bp)
Peristrophe japonica	151,374	83,395	17,073	25,453	80/30/4	38.1	36.1	32.1	43.3	*rps19* (102 bp)	Overlap 117 bp; ycf*1* (812 bp)
Pseuderanthemum haikangense	152,849	83,878	17,273	25,849	80/30/4	38.4	36.5	32.8	43.3	IGS (*rpl22/rps19*)	Overlap 121 bp; *ycf1* (816 bp)
Ruellia brittoniana	143,016	91,857	17,591	16,784	80/30/4	38.4	36.7	32.7	46	*ycf2* (433 bp)	Overlap 37 bp; ycf*1* (764 bp)
Rungia pectinata	149,627	81,979	16,626	25,511	80/30/4	38	36.1	32	43.1	*rps22* (339 bp)	Overlap 122 bp; *ycf1* (817 bp)
Staurogyne concinnula	153,783	84,636	17,855	25,646	80/30/4	38	36.1	32	43.3	*rps19* (56 bp)	Overlap 23 bp; *ycf1* (903 bp)

Species	Length (bp)	LSC length (bp)	SSC length (bp)	IR length (bp)	Gene content (Protein /tRNA/ rRNA)	CG content	CG content (LSC)	CG content (SSC)	CG content (IR)	Junction	
										LSC/IR	SSC/IR
Strobilanthes bantonensis	144,591	92,068	17,766	17,379	80/30/4	38.2	36.5	32.4	45.6	IGS (*ycf2/psbA*)	Overlap 44 bp; ycf*1* (771 bp)
Strobilanthes biocullata	144,012	91,268	17,666	17,539	80/30/4	38.2	36.6	32.4	45.6	IGS (*ycf2/psbA*)	Overlap 44 bp; *ycf1* (771 bp)
Strobilanthes crispa	144,987	92,556	17,783	17,324	80/30/4	38.2	36.5	32.6	45.6	IGS (*ycf2/psbA*)	*Overlap* 15 bp; *ycf1* (771 bp)
Strobilanthes cusia	144,133	91,666	17,811	17,328	80/30/4	38.2	36.5	32.4	45.7	IGS (*ycf2/psbA*)	*Overlap* 44 bp; *ycf1* (771 bp)
Strobilanthes medahinnensis	144,893	92,406	17,817	17,335	80/30/4	38.2	36.5	32.8	45.7	IGS (*ycf2/psbA*)	*Overlap* 44 bp; *ycf1 (771 bp)*
Strobilanthes tonkinensis	144,765	92,248	17,793	17,362	80/30/4	38.2	36.5	32.5	45.6	IGS (*ycf2/psbA*)	*Overlap* 44 bp; *ycf1 (771 bp)*
Thunbergia erecta	152,202	84,232	17,656	25,157	80/30/4	38.5	36.6	33.2	43.5	*rps19* (35 bp)	*Overlap* 67 bp; *ycf1 (762 bp)*
