# Supplementary material for: The complete chloroplast genome of Dicliptera tinctoria (Nees) Kostel. and comparative analysis of chloroplast genomes in Acanthaceae
Source: Genet Mol Biol. 2024 Jun 14;47(2):e20230297. doi: 10.1590/1678-4685-GMB-2023-0297 (PMC11182309; doi:10.1590/1678-4685-GMB-2023-0297)
Supplement: Figure S4 - [file 1415-4757-GMB-47-2-e20230297-s6.pdf]

Genetics and Molecular Biology
DOI: https://doi.org/10.1590/1678-4685-GMB-2023-0297

Supplementary Material to “The complete chloroplast genome of Dicliptera tinctoria (Nees) Kostel. and comparative analysis of chloroplast genomes in Acanthaceae”

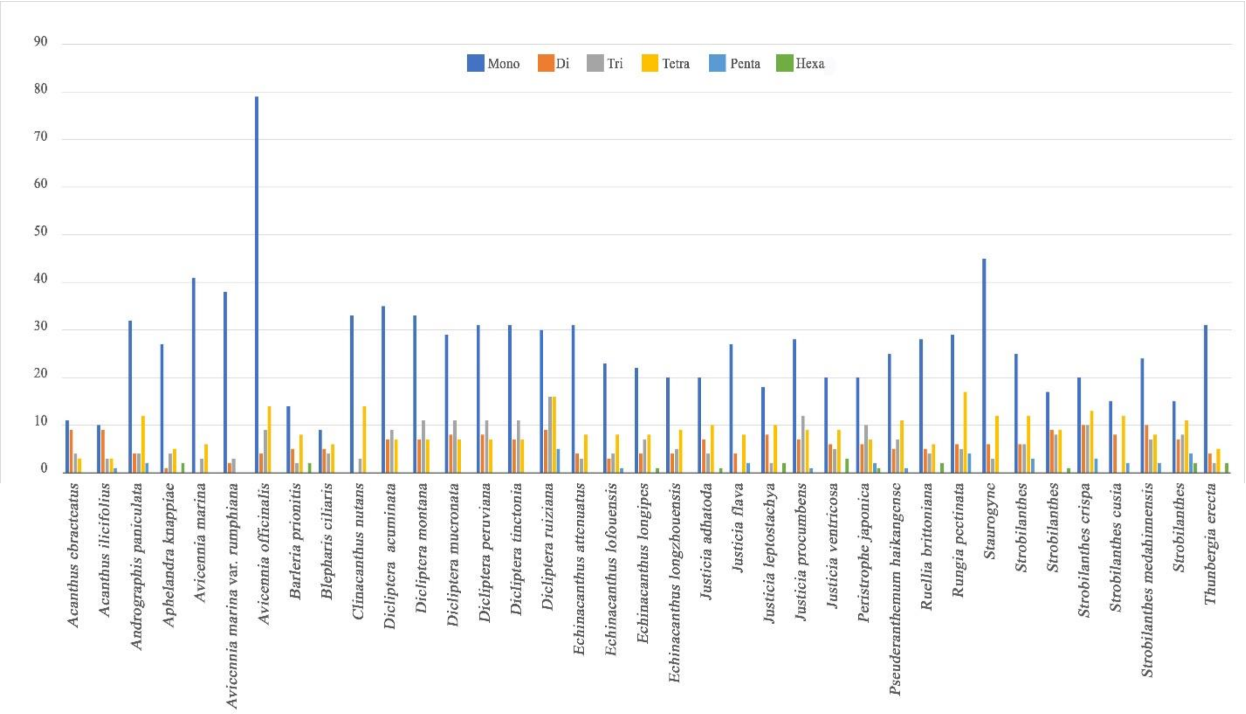

Figure S4 - The number of SSR found in chloroplast genomes of Acanthaceae species. The blue line revealed the total number of each species; the type of SSRs was represented by the color column on the right.
