# Supplementary material for: The complete chloroplast genome of Dicliptera tinctoria (Nees) Kostel. and comparative analysis of chloroplast genomes in Acanthaceae
Source: Genet Mol Biol. 2024 Jun 14;47(2):e20230297. doi: 10.1590/1678-4685-GMB-2023-0297 (PMC11182309; doi:10.1590/1678-4685-GMB-2023-0297)
Supplement: Figure S5 - [file 1415-4757-GMB-47-2-e20230297-s7.pdf]

*Genetics and Molecular Biology*
DOI: https://doi.org/10.1590/1678-4685-GMB-2023-0297

# Supplementary Material to "The complete chloroplast genome of Dicliptera tinctoria (Nees) Kostel. and comparative analysis of chloroplast genomes in Acanthaceae"

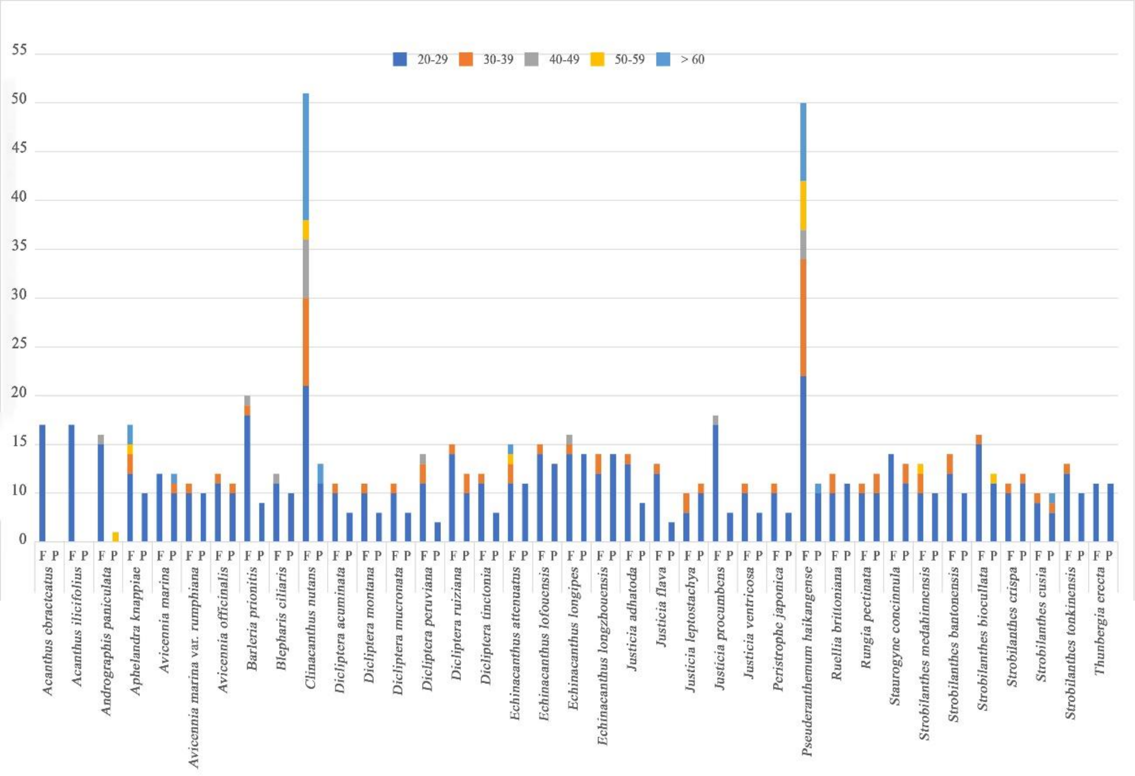


**Figure S5 -** The number of two types of long repeats among cpDNA of Acanthaceae. P stands for palindromic; F stands for forward; the green line represents the total number of both forward and palindromic repeats of Acanthaceae.
